# Association Between Herpes Simplex Virus Type 2 and High-Risk Human Papillomavirus Infections: A Population Study of the National Health and Nutrition Examination Survey, 2009–2016

**DOI:** 10.1093/infdis/jiaf033

**Published:** 2025-01-15

**Authors:** Chuqi Liu, Yongzhen Guo, Lulu Wang, Ruixia Guo, Dongmei Lei

**Affiliations:** Department of Obstetrics and Gynecology, Third Affiliated Hospital of Zhengzhou University, Zhengzhou University, Zhengzhou, Henan, China; Department of Pathology, Third Affiliated Hospital of Zhengzhou University, Zhengzhou University, Zhengzhou, Henan, China; Zhengzhou Key Laboratory of Gynecological Disease's Early Diagnosis, Third Affiliated Hospital of Zhengzhou University, Zhengzhou University, Zhengzhou, Henan, China; Department of Obstetrics and Gynecology, Third Affiliated Hospital of Zhengzhou University, Zhengzhou University, Zhengzhou, Henan, China; Department of Gynecology, First Affiliated Hospital of Zhengzhou University, Zhengzhou University, Zhengzhou, Henan, China; Department of Pathology, Third Affiliated Hospital of Zhengzhou University, Zhengzhou University, Zhengzhou, Henan, China; Zhengzhou Key Laboratory of Gynecological Disease's Early Diagnosis, Third Affiliated Hospital of Zhengzhou University, Zhengzhou University, Zhengzhou, Henan, China

**Keywords:** cervical cancer, herpes simplex virus type 2, high-risk human papillomavirus, National Health and Nutrition Examination Survey, sexually transmitted diseases

## Abstract

**Background:**

Human papillomavirus (HPV) is a leading cause of cervical cancer, with 14 subtypes classified as high-risk HPV (HR-HPV). Despite the availability of vaccines, certain regions still experience limited access. Herpes simplex virus type 2 (HSV-2), a common sexually transmitted infection, is hypothesized to increase the risk of HR-HPV infections. This study aims to individually analyze whether HSV-2 infection increases the risk of each HR-HPV infection in a representative sample of American adults.

**Methods:**

Data were derived from the National Health and Nutrition Examination Survey from 2009 to 2016, involving 4076 female participants. The study utilized logistic regression to estimate the link between HSV-2 infection and HR-HPV infection. We also conducted a stratified analysis to evaluate the impact of HSV-2 on HR-HPV infection in different subgroups.

**Results:**

After adjustment, the odds of having HR-HPV infection were 1.46 (95% CI, 1.24–1.71) for those with HSV-2 infection. Moreover, women with HSV-2 infection had higher odds of HPV-18 (odds ratio, 3.01; 95% CI, 2.05–4.41) and HPV-58 (OR, 2.05; 95% CI, 1.52–3.32) infection even after adjusting for potential confounding factors. The results remain significant in subgroup analysis and in the interaction test.

**Conclusions:**

The study found a significant association between HSV-2 infection and HR-HPV infection, particularly with HPV-18 and HPV-58, highlighting the importance of preventing HSV infection and advocating for early vaccination with an HPV vaccine for those vulnerable to HSV infection. Further prospective studies are needed to validate causal associations and elucidate the underlying mechanisms.

## Teaser

Herpes simplex virus type 2 infection raises the odds of high-risk human papillomavirus (HPV) infection, especially HPV-18 and HPV-58. This study underscores the importance of preventing herpes simplex virus and promoting early HPV vaccination among individuals at risk to reduce cervical cancer incidence.

Human papillomavirus (HPV) is one of the most common sexually transmitted infections, and persistent high-risk HPV (HR-HPV) infection is associated with cervical cancer [1]. More than 200 HPV subtypes have been found to infect humans, among which 14 have been identified as carcinogenic (HPV-16, 18, 31, 33, 35, 39, 45, 51, 52, 56, 58, 59, 66, and 68) [2]. While the introduction of HPV vaccines has significantly decreased the prevalence of cervical cancer, access to these vaccines remains limited in certain moderately developed countries and regions [3]. Given the substantial negative impact of HR-HPV on human health, identifying the risk factors for HR-HPV is crucial for distinguishing high-risk populations, taking timely preventive measures for such populations, and reducing the risk of cervical cancer.

Herpes simplex virus 2 (HSV-2), a member of the Herpesviridae family along with HSV-1, possesses a linear double-stranded DNA genome enclosed within an icosahedral capsid [4]. The transmission of HSV-2 occurs primarily through sexual contact, resulting in its widespread global distribution [5]. Current estimates suggest that as of 2016, approximately 491.5 million individuals worldwide, constituting roughly 13% of the population, have been infected with HSV-2 [6, 7]. HSV-2 is typically transmitted through viral shedding via genital contact and is considered the main cause of sexually transmitted infections [8]. Following initial infection, the virus establishes lifelong persistence within the host, leading to the development of oral and genital ulcers, herpetic keratitis, and encephalitis [9].

A study conducted by researchers at Peking University First Hospital found a significant association between sexually transmitted infections and HPV infection in individuals with cervical cancer, noting that those who tested positive for HSV-2 had a higher risk of HPV infection [10]. Nevertheless, the research conducted so far has a limited sample size, and there is a scarcity of studies examining the relationship between HSV-2 and subtypes of HR-HPV. The primary objective of this study is to investigate the association between HSV-2 and HR-HPV subtypes in a large, nationally representative sample of American adults, with the intention of determining whether HSV-2 infection serves as a risk factor for HR-HPV infection and examining the potential link between HSV-2 infection and cervical cancer.

## METHODS

### Study Design and Population

The National Health and Nutrition Examination Survey (NHANES) is an annual survey conducted by the National Center for Health Statistics, a subsidiary of the Centers for Disease Control and Prevention in the United States. It aims to collect data from nationally representative samples of American civilians and noninstitutional individuals. This survey needs to be reviewed and approved by the Disclosure Review Committee of the National Center for Health Statistics. Further information on ethical approval and informed consent procedures can be obtained from the National Center for Health Statistics [11]. Comprehensive information on the design, methodology, and weighting of the NHANES has been published [11, 12]. From 2009 to 2016, the NHANES employed a stratified, complex, multistage sampling methodology. All data are available through the NHANES website, and sample weights are used to account for participant selection probability and nonresponse adjustments. The survey's structure and weighting formula have been discussed [11], and this report adheres to the STROBE (Strengthening the Reporting of Observational Studies in Epidemiology) reporting guidelines for cross-sectional studies.

**Figure 1.**
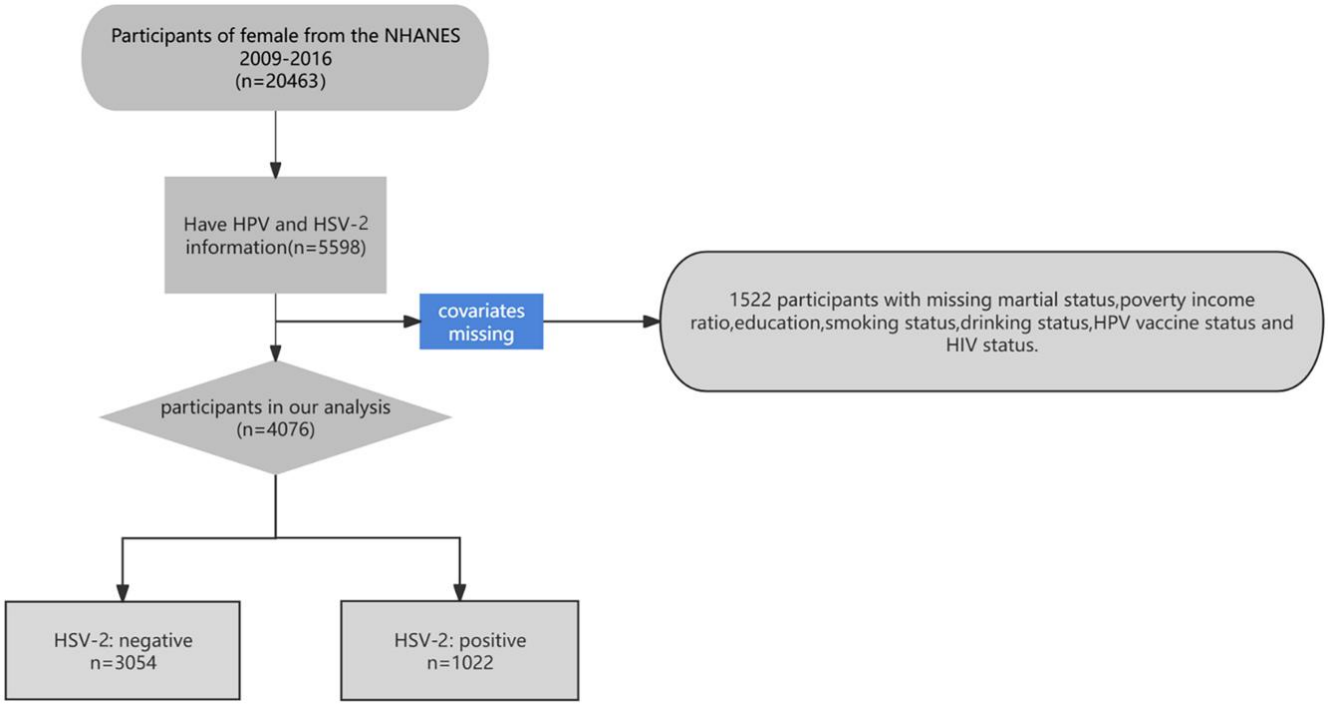
Flowchart of the population included in the final analysis. HPV, human papillomavirus; HSV-2, herpes simplex virus 2; NHANES, National Health and Nutrition Examination Survey.

In this study, data were employed from all 4 available survey cycles (2009–2016), which contain 20 463 US female participants. Since public records for genital HPV infection and status of HSV-2 infection are subject to data restrictions, our analysis included individuals who were ≥18 years old but <49 years at the time of participation and completed the required tests. After exclusion of those with missing (n = 12 854) or indeterminate (n = 44) HPV measurements, 7565 females were included in the analysis. We also excluded persons with missing HSV-2 measurements (n = 1967), leaving 5598 female participants. We sequentially excluded 839 participants with missing demographic data, including marital status, education status, and poverty income ratio (PIR); 531 without drinking status; 2 without smoking status; 147 without HPV vaccine status; and 3 without HIV infection status. Consequently, 16 387 participants were excluded from the study, leaving a final analytic sample size of 4076 (Figure 1).

### Detection of HPV Infection

NHANES Mobile Examination Center (MEC) personnel explained the process for self-collection of vaginal samples to participants and provided an individually packaged Dacron swab (Catch-All; Epicentre) in a transport sleeve. Vaginal and cervical exfoliated cells were collected by research participants by inserting the swab vaginally, similar to tampon insertion, and rotating as directed. Participants then placed the swab into the sleeve and returned it to NHANES personnel. HPV genotyping was achieved with the Linear Array HPV Genotyping Test. If participants had any of the 14 HR-HPV genotypes (16, 18, 31, 33, 35, 39, 45, 51, 52, 56, 58, 59, 66, or 68), they were considered to have an HR-HPV infection. Participants who tested positive for β-globin controls but had no infection of the aforementioned HPV types were considered negative for HR-HPV infection. Samples that tested negative for HR-HPV and had negative β-globin controls were unsuitable for analysis, while participants who did not provide or had inadequate cervicovaginal samples were excluded from analysis. The detailed methodology is available in the NHANES laboratory procedure manual [13].

### Detection of HSV Infection

Blood samples were collected from eligible participants at a MEC and shipped to Emory University or the Centers for Disease Control and Prevention for HSV analysis. The serostatus of HSV-1 and HSV-2 was accessed by a solid-phase enzymatic immune dot assay with a virus type–specific purified glycoprotein as antigen, and the outcomes were categorized as positive, negative, and indeterminate.

### Covariates

Potential covariates were selected for clinical relevance and statistical significance as follows: demographic data (age, race and ethnicity, PIR, educational level, and marital status), lifestyle variables (smoking status and alcohol drinking), immunization data (HPV vaccine status), and laboratory results (HIV infection). Demographic, lifestyle, and immunization data were derived from the household interview questionnaires administered by highly trained medical personnel. Anthropometric indicators and biochemical parameters were obtained through medical examinations and subsequent laboratory assessments in the MEC.

Educational level was categorized as grade <9, grades 9 to 11, high school graduate, some college or an associate of arts degree, and college graduate or above. Race and ethnicity were classified as Mexican American, other Hispanic, non-Hispanic Black, non-Hispanic White, and other. Smoking status was determined by “smoked at least 100 cigarettes in life,” and alcohol drinking status was evaluated by “had at least 12 alcohol drinks per year.” Cancer status was determined by questions in the medical condition questionnaire: “Have you ever been told by a doctor or other health professional that you have cancer or any type of malignant tumor?” and “What type of cancer is it?” This study includes data on cervical cancer.

### Statistical Analysis

Four waves of continuous survey data were combined (NHANES 2009–2010, 2011–2012, 2013–2014, and 2015–2016), and an 8-year sampling weight was computed by utilizing a quarter of the 2-year sampling weight. Participants were categorized into 2 groups according to their HSV-2 infection status. Data are displayed as weighted mean ± SD for continuous variables and frequency (weighted percentage) for categorical variables. For comparisons between the groups, *t* tests for normally distributed data, Mann-Whitney tests for skewed distributions, or χ^2^ tests for categorical variables were employed as needed. The use of weighted multiple logistic regression models formed the basis for examining the association between HR-HPV infection and HSV infection. At the same time, the correlation between HSV-2 and 14 high-risk types was examined one by one, along with the association between HSV-2 and the occurrence of cervical cancer. Additional stratified analyses were conducted, categorizing participants according to various factors such as age, body mass index, PIR, alcohol intake, smoking habits, education, marital status, and HPV vaccine status. Three models were assessed:

Crude model: no adjustment

Model 1: adjusted for age, race, PIR, education level, alcohol consumption and smoking status, and history of receiving HPV vaccine

Model 2: covariate adjustments encompassed age, race, PIR, education level, marital status, alcohol consumption, smoking status, and history of receiving HPV vaccine

All analytic processes were conducted with R package version 3.4.3 and EmpowerStats version 4.1. A significance threshold of *P* < .05 guided the determination of statistical significance. Weight calculation for the 4 survey cycles was accomplished by dividing the MEC examination weights (WTMEC2YR) by 4.

## RESULTS

### Baseline Characteristics of Study Participants

This study involved 4076 female participants, with an average age of 32 years. Among these women, 31.91% were infected with HR-HPV. Participants were categorized into 2 groups by HSV-2 status: negative and positive. A detailed summary of weighted baseline characteristics is described in Table 1: women who tested positive for HSV-2 tended to be older (38.13 vs 33.86 years), were more likely to be non-Hispanic Black (29.53% vs 7.58%), had lower PIRs (2.35 vs 2.89), had lower levels of education (41.7% vs 26.48%), were less likely to be married (40.21% vs 53.25%), and had higher rates of cigarette and alcohol consumption. When compared with the HSV-2–negative group, the HSV-2–positive group had a higher proportion of individuals who tested positive for HR-HPV (27.47% vs 21.34%). The number of persons infected for each of the 14 HR-HPV types is described in Supplementary Table 2.

**Table 1.** Weighted Characteristics of the Study Population by HSV-2 Infection Status

	Total (N = 4076)		HSV-2 Negative (n = 3054)		HSV-2 Positive (n = 1022)		
Characteristic	No.	Weighted Mean (95% CI)	No.	Weighted Mean (95% CI)	No.	Weighted Mean (95% CI)	*P* Value
Age at screening, y	4076	34.76 (34.32–35.21)	3054	33.86 (33.29–34.43)	1022	38.13 (37.40–38.87)	<.0001
Body mass index, kg/m^2^	4065	28.94 (28.62–29.26)	3046	28.37 (28.04–28.71)	1019	31.06 (30.39–31.72)	<.0001
Poverty income ratio	4076	2.78 (2.67–2.89)	3054	2.89 (2.78–3.01)	1022	2.35 (2.19–2.50)	<.0001
Age at menarche, y	4053	12.64 (12.58–12.71)	3035	12.65 (12.58–12.73)	1019	12.63 (12.52–12.74)	.6647
No. of pregnancies	3057	2.83 (2.75–2.91)	2154	2.70 (2.61–2.79)	915	3.29 (3.16–3.42)	<.0001
Vaginal deliveries	3048	1.58 (1.52–1.65)	2140	1.53 (1.45–1.62)	911	1.73 (1.64–1.83)	.0064

		Weighted % (95% CI)		Weighted % (95% CI)		Weighted % (95% CI)	
Ever been pregnant							<.0001
No	995	27.30 (24.62–30.17)	889	31.23 (27.95–34.71)	106	12.77 (10.07–16.07)	
Yes	3053	72.70 (69.83–75.38)	2139	68.77 (65.29–72.05)	914	87.23 (83.93–89.93)	
Race							<.0001
Mexican American	642	10.02 (7.82–12.74)	540	10.67 (8.24–13.70)	102	7.59 (5.69–10.05)	
Other Hispanic	414	6.66 (5.28–8.37)	299	6.35 (4.97–8.08)	115	7.81 (5.74–10.54)	
Non-Hispanic White	1646	62.67 (58.29–66.84)	1332	66.14 (61.76–70.27)	314	49.70 (43.70–55.71)	
Non-Hispanic Black	820	12.14 (10.10–14.53)	398	7.48 (6.12–9.11)	422	29.53 (25.05–34.45)	
Other	554	8.52 (7.56–9.58)	485	9.36 (8.23–10.63)	69	5.37 (3.99–7.21)	
Education level							<.0001
Grade <9	205	3.29 (2.62–4.13)	141	2.87 (2.19–3.75)	64	4.86 (3.69–6.40)	
Grades 9–11	480	9.07 (7.98–10.28)	312	7.81 (6.73–9.04)	168	13.75 (11.50–16.36)	
High school graduate	758	17.34 (15.60–19.24)	526	15.80 (14.12–17.65)	232	23.09 (19.51–27.10)	
Some college or associate degree	1454	36.10 (34.10–38.16)	1060	35.31 (33.07–37.61)	394	39.08 (34.94–43.39)	
College graduate or above	1179	34.20 (31.29–37.22)	1015	38.21 (34.97–41.57)	164	19.22 (15.67–23.35)	
Marital status							<.0001
Married	1874	50.49 (48.15–52.83)	1526	53.25 (50.66–55.82)	348	40.21 (36.29–44.26)	
Widowed	35	0.89 (.57–1.38)	13	0.41 (.20–.83)	22	2.68 (1.48–4.81)	
Divorced	361	8.79 (7.69–10.03)	216	7.30 (6.18–8.59)	145	14.38 (11.72–17.52)	
Separated	155	2.94 (2.42–3.58)	91	2.45 (1.90–3.15)	64	4.79 (3.67–6.22)	
Never married	1167	25.23 (22.80–27.83)	853	25.11 (22.40–28.02)	314	25.69 (22.35–29.35)	
Living with partner	484	11.65 (10.50–12.92)	355	11.49 (10.20–12.92)	129	12.26 (10.15–14.73)	
Alcohol drinks							.8720
No	1245	24.18 (21.87–26.65)	936	24.25 (21.63–27.09)	309	23.92 (20.52–27.68)	
Yes	2831	75.82 (73.35–78.13)	2118	75.75 (72.91–78.37)	713	76.08 (72.32–79.48)	
Smoke							<.0001
No	2736	63.79 (61.32–66.20)	2178	67.66 (64.94–70.26)	558	49.40 (45.09–53.72)	
Yes	1340	36.21 (33.80–38.68)	876	32.34 (29.74–35.06)	464	50.60 (46.28–54.91)	
Received HPV vaccine							<.0001
No	3531	86.00 (84.31–87.53)	2598	84.41 (82.33–86.28)	933	91.93 (89.93–93.57)	
Yes	545	14.00 (12.47–15.69)	456	15.59 (13.72–17.67)	89	8.07 (6.43–10.07)	
HIV status							.0040
Negative	4068	99.85 (99.70–99.93)	3052	99.94 (99.74–99.99)	1016	99.54 (98.97–99.80)	
Positive	8	0.15 (.07–.30)	2	0.06 (.01–.26)	6	0.46 (.20–1.03)	
High-risk HPV status							.0006
Negative	3084	77.36 (75.63–79.01)	2366	78.66 (76.76–80.43)	718	72.53 (69.05–75.76)	
Positive	992	22.64 (20.99–24.37)	688	21.34 (19.57–23.24)	304	27.47 (24.24–30.95)	

Data for continuous variables: observed number and survey-weighted mean (95% CI); *P* value by survey-weighted linear regression. Data for categorical variables: observed number and survey-weighted percentage (95% CI); *P* value by survey-weighted χ^2^ test.

Abbreviations: HPV, human papillomavirus; HSV-2, herpes simplex virus type 2.

### Association Between HSV-2 and HR-HPV

We examined the association between HSV-2 infection and HR-HPV infection through logistic regression analysis (Table 2). Crude logistic regression showed that HSV-2 seropositivity was associated with HR-HPV (odds ratio [OR], 1.46; 95% CI, 1.24–1.71), indicating that HSV-2 is a notable risk factor for HR-HPV infection. After adjusting for covariates in different models, the OR was slightly reduced but remained statistically significant (OR, 1.38 [95% CI, 1.15–1.66] in model 1 and 1.27 [95% CI, 1.06–1.54] in model 2).

**Table 2.** Crude and Adjusted Associations Between HSV-2 Infection and High-Risk HPV Infection

	Crude		Model 1^a^		Model 2^b^	
HPV Outcome: HSV-2 Infection Status	OR (95% CI)	*P* Value	OR (95% CI)	*P* Value	OR (95% CI)	*P* Value
High-risk HPV		<.0001		.0006		.0114
Negative	1 [Reference]		1 [Reference]		1 [Reference]	
Positive	1.46 (1.24–1.71)		1.38 (1.15–1.66)		1.27 (1.06–1.54)	
HPV-58		.0005		.0024		.0071
Negative	1 [Reference]		1 [Reference]		1 [Reference]	
Positive	2.20 (1.41–3.44)		2.24 (1.33–3.77)		2.06 (1.22–3.48)	
HPV-18		<.0001		.0002		.0007
Negative	1 [Reference]		1 [Reference]		1 [Reference]	
Positive	3.47 (2.25–5.35)		2.52 (1.54–4.12)		2.36 (1.44–3.87)	
HPV-16/18		.0036		.0403		.1740
Negative	1 [Reference]		1 [Reference]		1 [Reference]	
Positive	1.51 (1.14–1.99)		1.39 (1.01–1.90)		1.25 (.91–1.71)	

Abbreviations: HPV, high risk-human papillomavirus; HSV-2, herpes simplex virus type 2; OR, odds ratio.

^a^Model 1 was adjusted for age, race, poverty income ratio, education level, had at least 12 alcohol drinks, smoked at least 100 cigarettes in life, received HPV vaccine, and HIV status.

^b^Model 2 was further adjusted for marital status.

Moreover, the association between HSV-2 infection status and each HR-HPV genotype was further analyzed. As depicted in Table 2, after adjusting for all confounding factors, the findings suggest that HSV-2 infection raises the risk of HPV-18 infection (OR, 3.01; 95% CI, 2.05–4.41; *P* < .0001). Additionally, HSV-2 infection is linked to an increased risk of HPV-58 infection (OR, 2.05; 95% CI, 1.52–3.32; *P* < .0001). HPV-16 and HPV-18 are the primary causes of cervical cancer, accounting for 70% of all cases [14]. Accordingly, we also investigated the connection between HSV infection and HPV-16/18 infection. The results indicated a statistically significant relationship between HSV-2 infection and HPV-16/18 infection in the unadjusted confounding model and model 1. However, in model 2, where confounding factors such as marital status were included, their association was no longer statistically significant. Our study did not find any association between HSV-2 and other HR-HPV infections (Supplementary Table 1).

### Stratified Analyses Based on Additional Variables

**Figure 2.**
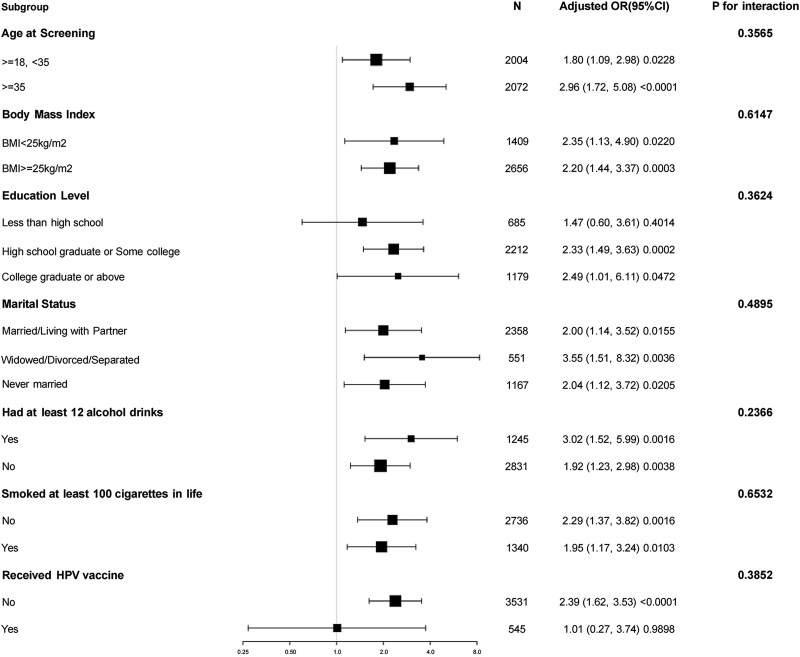
The relationship between HSV-2 infection and HPV-18 or HPV-58 infection according to basic features. Except for the stratification component itself, each stratification factor was adjusted for all other variables: age, race and ethnicity, education, marital status, family income, alcohol, smoke, HPV vaccine status, HIV status. HPV, human papillomavirus; HSV-2, herpes simplex virus 2; OR, odds ratio.

We conducted a stratified analysis to evaluate the impact of HSV-2 on HPV-18/58 infection in different subgroups (Figure 2). A significant association was observed between HSV-2 and HPV-18/58 infection among individuals aged >35 years (OR, 2.96; 95% CI, 1.72–5.08), those with higher education (OR, 2.49; 95% CI, 1.01–6.11), those who are widowed/divorced/separated (OR, 3.55; 95% CI, 1.51–8.32), those who consume alcohol (OR, 3.02; 95% CI, 1.52–5.99), and those who have not received the HPV vaccine (OR, 2.39; 95% CI, 1.62–3.53). No significant interactions were observed in any subgroup. The results of the interaction test indicated that the differences among subgroups did not reach a statistically significant level, suggesting that these variables did not significantly modify the association between HSV-2 infection and HPV-18/58 infection. This implies that the link between HSV-2 infection and HPV-18/58 infection remains relatively stable.

### Association Between HSV-2 and Cervical Cancer

In the subsequent analysis, the relationship between HSV-2 and the prevalence of cervical cancer was investigated with data from NHANES 2009 to 2016 (n = 3966; Table 3). When compared with the HSV-2–negative group, the HSV-2–positive group had a higher proportion of individuals who had cervical cancer (2.63% vs 0.91%; Supplementary Table 3). Crude logistic regression indicated that HSV-2 seropositivity was associated with the occurrence of cervical cancer (OR, 2.96; 95% CI, 1.72–5.09; *P* < .0001). In model 1 (adjusted for some confounders), a connection between HSV-2 seropositivity and cervical cancer was established (adjusted OR, 2.40; 95% CI, 1.31–4.40; *P* = .0045). In model 2, HSV-2 remained associated with cervical cancer even after additional adjustment for HPV infection status as a confounder (OR, 2.42; 95% CI, 1.32–4.44; *P* = .0043).

**Table 3.** Associations Between HSV-2 and Cervical Cancer per NHANES Data, 2009–2016

		Crude		Model 1^a^		Model 2^b^	
Exposure	No. (%)	OR (95% CI)	*P* Value	OR (95% CI)	*P* Value	OR (95% CI)	*P* Value
HSV-2			<.0001		.0045		.0043
Negative	2979 (75.11)	1 [Reference]		1 [Reference]		1 [Reference]	
Positive	987 (24.89)	2.96 (1.72–5.09)		2.40 (1.31–4.40)		2.42 (1.32–4.44)	

Abbreviations: HPV, human papillomavirus; HSV-2, herpes simplex virus type 2; NHANES, National Health and Nutrition Examination Survey; OR, odds ratio.

^a^Model 1 was adjusted for age, race, poverty income ratio, education level, marital status, had at least 12 alcohol drinks, smoked at least 100 cigarettes in life, received HPV vaccine, and HIV status.

^b^Model 2 was further adjusted for high-risk HPV status.

## DISCUSSION

To clarify the connection between HSV-2 infection and HR-HPV infection, a cross-sectional analysis was performed on 4076 individuals from NHANES. The findings indicate that persons with HSV-2 infection are at a higher risk of being infected with high-risk HPV-18/58. Furthermore, this correlation was consistent in stratified analysis.

Recent research has discovered a possible connection between HSV-2 infection and HR-HPV infection. A cross-sectional study explored the correlation between common sexually transmitted infections and HPV infection, recruiting patients from the gynecology outpatient department of Peking University First Hospital [10]. They discovered that the risk of HPV infection in the HSV-2–positive group was significantly higher than that in the HSV-2–negative group (OR, 11.032; 95% CI, 1.465–83.056; *P* = .020). In our research, we conducted an analysis on the relationship between HSV-2 and HR-HPV (16, 18, 31, 33, 35, 39, 45, 51, 52, 56, 58, 59, 66, or 68), revealing that HSV-2 infection raises the risk of HR-HPV infection (OR, 1.27; 95% CI, 1.06–1.54). The research also indicated that the correlation between HPV-16/18 infection and HSV-2 infection status was not statistically significant [10], aligning with our research conclusion. However, this study has limitations. First, the sample size was small, with only 19 cases of HSV-2 infection, of which there were 18 cases of HR-HPV infection. Additionally, the analysis adjusted for a limited number of confounding factors, adjusting only for age and aerobic vaginitis/desquamative inflammatory vaginitis. In contrast, our study utilized the NHANES database and made adjustments for potential confounding factors as comprehensively as possible.

However, research has shown that HSV-2 infection is linked to a higher likelihood of HPV-16/18 infection. Using NHANES data from 2003 to 2010, Li and Wen discovered that individuals with HSV-2 seropositivity face an elevated risk of HPV-16/18 infection as compared with those with HSV-2 negativity [15]. This is inconsistent with our study, possibly because the variables controlled for in this study did not encompass marital status. Our research revealed a notable difference in marital status distribution between people with and without HR-HPV infection. Our study also indicated a higher risk of high-risk HPV-16/18 infection in persons with HSV-2 seropositivity in unadjusted and partially adjusted models. Nevertheless, upon adjusting for all variables (model 2), including marital status, we observed no statistically significant association between high-risk HPV-16/18 infection and HSV-2 infection.

Moreover, there is limited research on the correlation between HSV-2 and HR-HPV. Additionally, the most prevalent types of HR-HPV infection are HPV-16 and HPV-18 [16], and most studies typically focus on HPV-16 and HPV-18. However, the connection between other high-risk types and cervical cancer is also significant. Our research involved logistic regression analysis on all 14 high-risk types, revealing a notable increase in the risk of HPV-18 (OR, 3.01) or HPV-58 (OR, 2.05) infection among individuals with HSV-2 seropositivity. Yet, no link has been observed between HSV-2 infection and other HR-HPVs. This discrepancy may be attributed to variations in genomic organization among these HPV types, leading to differences in their infection mechanisms. Therefore, further study is needed to delve into how HSV-2 infection specifically affects the infection and pathogenic processes of different HPV types.

HSV-2 and HPV are lifelong infections affecting genital sites, and they share similar risk factors, such as sexual behavior, which increases the risk of spurious associations in observational studies due to confounding. We minimized the risk of confounding at the study level by focusing our analysis on adjusted estimates. Some studies have found that individuals infected with HIV are more susceptible to HPV and HSV-2 infection [17]. The infection rates of HSV-2, HPV, Epstein-Barr virus, and squamous intraepithelial lesion in women who were HIV positive (75.2%, 41.9%, 41%, and 32.4%) were higher than they were in women who were HIV negative (45.7%, 26.7%, 26.7%, and 13.3%), with *P* < .0001, *P* = .029, *P* = .041, and *P* = .002, respectively [18]. HIV is also significantly associated with HSV-2 infection. More than half of people with HIV are coinfected with HSV-2, and the risk of HIV infection almost triples in those infected with HSV-2 [19, 20]. Therefore, in analyzing the relationship between HSV-2 infection and HPV infection, our study adjusted for the HIV infection status of participants as a confounding factor. After adjusting for HIV status, our conclusion still holds.

To verify the stability of the results, stratified analysis and an interaction effect test were conducted on factors such as age, body mass index, marital status, education level, smoking and alcohol consumption, and whether HPV vaccine was administered. The results remained consistent. No significant interactions were found in any subgroup. This implies that the link between HSV-2 infection and HPV-18/58 infection remains relatively stable.

There is a strong link between HR-HPV infection and the onset of cervical cancer. Nevertheless, only a small number of women with HR-HPV will actually develop cervical cancer, suggesting that other factors must play a role in the development of cervical cancer [21]. Meanwhile, in the 1980s, McDougall et al discovered HSV-2 RNA in the cells of precancerous lesions; however, there was no evidence to indicate the presence of HSV-2 RNA in the cells of squamous cell carcinoma [22]. Now, various studies are investigating the connection between HSV-2 and cervical cancer, and the outcomes of these studies are varied. Our results align with several other studies that have indicated a relationship between HSV-2 and cervical cancer. According to the NHANES data from 2003 to 2010, Li and Wen showed that HSV-2 infection was significantly associated with cervical cancer [15]. Yet, they did not account for the confounding factor of marital status in their analysis. Our study utilized NHANES data from 2009 to 2016 and further adjusted for marital status as a confounding factor. Notably, Zhang et al found a significant positive correlation between HSV-2 infection and cervical cancer (OR, 3.01; 95% CI, 2.24–4.04) and precancerous lesions (OR, 2.14; 95% CI, 1.55–2.96). This correlation may due to HSV-2 infection causing instability in the host genome, as well as the inflammation triggered by its long-term latent infection, thereby increasing the risk of carcinogenesis [23]. Still, some studies have shown little correlation between HSV-2 and cervical carcinogenesis. In a study on women in Nordic countries, Lehtinen concluded that HSV-2 was not linked to the pathogenesis of cervical cancer [24], and a study evaluating Iranian women discovered just a single instance of HSV-2 infection among 45 patients with cervical cancer [25]. It is important to note that the sample sizes in these studies were relatively small.

Although the precise mechanism of the positive correlation between HSV-2 and HR-HPV infection remains unclear, our findings are biologically plausible according to current evidence. One possible mechanism is the development of ulcers due to HSV-2 infection, which could aid in the penetration of HPV into basal cells [26, 27]. Moreover, this inflammation may hinder an effective immune response against HPV infection by suppressing T-helper–mediated immunity and triggering nitric acid production, potentially causing cellular DNA damage in HPV-infected cells and increasing the risk of carcinogenesis.

Our findings suggest that due to the strong association between HPV infection and HSV-2, it is necessary to strengthen the prevention of HSV-2. Additionally, for populations infected with HSV-2, it is essential to increase the uptake of HPV vaccines, particularly those targeting HPV-18 and HPV-58, and enhance cervical cancer screening efforts. However, several limitations of this study should be noted: the cross-sectional design limited causal inference; potential unmeasured confounders may exist despite our comprehensive adjustments; and the generalizability of the findings is limited to US women aged 18 to 49 years. Future prospective studies and mechanistic research are needed to validate these associations and expand the findings to broader populations.

## CONCLUSION

This study uncovers a connection between HSV-2 infection and HR-HPV infection in women aged 18 to 49 years in the United States. Specifically, HSV-2 infection may elevate the risk of high-risk HPV-18/58 infection among 14 types, even after considering potential confounding factors. The results of subgroup stratification analysis support this association. The research findings underscore the importance of preventing HSV infection and propose that individuals infected with HSV should receive the HPV vaccine (Gardasil 9) as early as possible.

## Supplementary Material

jiaf033_Supplementary_Data

## Supplementary Data

Supplementary materials are available at *The Journal of Infectious Diseases* online (http://jid.oxfordjournals.org/). Supplementary materials consist of data provided by the author that are published to benefit the reader. The posted materials are not copyedited. The contents of all supplementary data are the sole responsibility of the authors. Questions or messages regarding errors should be addressed to the author.

## Notes

***Acknowledgments.*** We appreciate the work by NHANES collaborators, and we are grateful to all the participants for their cooperation to make this study possible.

***Author contributions.*** All authors contributed to study conception and design. C. L. and Y. G. contributed equally to this work. C. L. and L. W. were responsible for data collection and analysis. C. L. and Y. G. conducted statistical methods and analysis and verification of data. C. L. was responsible for interpreting the results and drafting the article. D. L. and Y. G. were responsible for revising it critically for important intellectual content. All authors read and approved the final manuscript.

***Data availability.*** The data used in this study are from NHANES (2009–2016). Data are publicly available and can be downloaded from the NHANES website: https://www.cdc.gov/nchs/nhanes/index.html.

***Ethics approval and consent to participate.*** Data analyzed in this study were obtained from NHANES. Protocols involved were approved by the National Center for Health Statistics Research Ethics Review Board, and consent from all participants was documented. This study is a secondary data analysis based on existing, deidentified, publicly available NHANES data, and no further ethical approval is required according to US Health and Human Services regulations (http://www.hhs.gov/ohrp/policy/cdebiol.html).

***Financial support.*** This work was supported by the Henan Province Science and Technology Research Project (grant SBGJ202302074 to R. G.).
