# Supplementary material for: Association Between Herpes Simplex Virus Type 2 and High-Risk Human Papillomavirus Infections: A Population Study of the National Health and Nutrition Examination Survey, 2009–2016
Source: J Infect Dis. 2025 Jan 15;231(4):e650–8. doi: 10.1093/infdis/jiaf033 (PMC11998555; doi:10.1093/infdis/jiaf033)
Supplement: jiaf033_Supplementary_Data [file jiaf033_supplementary_data.zip › Additional-file-1-tableS1.docx]

**Additional file 1:**

Table S1: Crude and adjusted association between HSV-II infection and HR-HPV infection.

|  | **Crude** | **Model 1** | **Model 2** |
| --- | --- | --- | --- |
|  | **OR (95%CI) P-value** | | |
| **Outcome: HPV type 16** |  |  |  |
| Herpes Simplex Virus II |  |  |  |
| Negative | 1.0(Ref) | 1.0(Ref) | 1.0(Ref) |
| Positive | 0.95 (0.7, 1.4) 0.769 | 1.05 (0.7, 1.6) 0.827 | 0.90 (0.6, 1.4) 0.620 |
| **Outcome: HPV type 31** |  |  |  |
| Herpes Simplex Virus II |  |  |  |
| Negative | 1.0(Ref) | 1.0(Ref) | 1.0(Ref) |
| Positive | 1.2 (0.7, 2.1) 0.420 | 1.3 (0.7, 2.3) 0.430 | 1.2 (0.6, 2.1) 0.619 |
| **Outcome: HPV type 33** |  |  |  |
| Herpes Simplex Virus II |  |  |  |
| Negative | 1.0(Ref) | 1.0(Ref) | 1.0(Ref) |
| Positive | 1.1 (0.5, 2.6) 0.754 | 0.8 (0.3, 1.9) 0.543 | 0.8 (0.3, 1.9) 0.579 |
| **Outcome: HPV type 35** |  |  |  |
| Herpes Simplex Virus II |  |  |  |
| Negative | 1.0(Ref) | 1.0(Ref) | 1.0(Ref) |
| Positive | 1.2 (0.7, 1.8) 0.550 | 1.0 (0.6, 1.7) 0.965 | 1.0 (0.6, 1.7) 0.922 |
| **Outcome: HPV type 39** |  |  |  |
| Herpes Simplex Virus II |  |  |  |
| Negative | 1.0(Ref) | 1.0(Ref) | 1.0(Ref) |
| Positive | 0.9 (0.6, 1.4) 0.651 | 1.3 (0.8, 2.1) 0.353 | 1.1 (0.7, 1.9) 0.598 |
| **Outcome: HPV type 45** |  |  |  |
| Herpes Simplex Virus II |  |  |  |
| Negative | 1.0(Ref) | 1.0(Ref) | 1.0(Ref) |
| Positive | 1.9 (1.2, 3.0) 0.004 | 1.6 (1.0, 2.7) 0.060 | 1.5 (0.9, 2.4) 0.151 |
| **Outcome: HPV type 51** |  |  |  |
| Herpes Simplex Virus II |  |  |  |
| Negative | 1.0(Ref) | 1.0(Ref) | 1.0(Ref) |
| Positive | 0.9 (0.6, 1.3) 0.543 | 1.0 (0.6, 1.5) 0.959 | 1.0 (0.6, 1.5) 0.866 |
| **Outcome: HPV type 52** |  |  |  |
| Herpes Simplex Virus II |  |  |  |
| Negative | 1.0(Ref) | 1.0(Ref) | 1.0(Ref) |
| Positive | 1.0 (0.7, 1.5) 0.979 | 1.0 (0.6, 1.5) 0.901 | 0.9 (0.6, 1.4) 0.734 |
| **Outcome: HPV type 56** |  |  |  |
| Herpes Simplex Virus II |  |  |  |
| Negative | 1.0(Ref) | 1.0(Ref) | 1.0(Ref) |
| Positive | 1.3 (0.8, 2.1) 0.255 | 1.6 (1.0, 2.9) 0.073 | 1.5 (0.8, 2.6) 0.169 |
| **Outcome: HPV type 59** |  |  |  |
| Herpes Simplex Virus II |  |  |  |
| Negative | 1.0(Ref) | 1.0(Ref) | 1.0(Ref) |
| Positive | 1.1 (0.7, 1.6) 0.799 | 1.1 (0.7, 1.7) 0.800 | 1.0 (0.6, 1.6) 0.986 |
| **Outcome: HPV type 66** |  |  |  |
| Herpes Simplex Virus II |  |  |  |
| Negative | 1.0(Ref) | 1.0(Ref) | 1.0(Ref) |
| Positive | 1.1 (0.7, 1.6) 0.719 | 1.1 (0.7, 1.8) 0.632 | 1.1 (0.7, 1.8) 0.737 |
| **Outcome: HPV type 68** |  |  |  |
| Herpes Simplex Virus II |  |  |  |
| Negative | 1.0(Ref) | 1.0(Ref) | 1.0(Ref) |
| Positive | 1.5 (0.9, 2.5) 0.083 | 0.9 (0.5, 1.7) 0.845 | 0.9 (0.5, 1.6) 0.666 |

Abbreviation: HR-HPV, high risk-human papillomavirus; OR, odds ratio; CI, confidence interval; Ref: reference.

Model 1 was adjusted for age; Race; PIR; Education Level; Had at least 12 alcohol drinks; Smoked at least 100 cigarettes in life; receive HPV vaccine; HIV status.

Model 2 was adjusted for age; Race; PIR; Education Level; Had at least 12 alcohol drinks; Smoked at least 100 cigarettes in life; receive HPV vaccine; HIV status; Marital Status;
