# Supplementary material for: Association Between Herpes Simplex Virus Type 2 and High-Risk Human Papillomavirus Infections: A Population Study of the National Health and Nutrition Examination Survey, 2009–2016
Source: J Infect Dis. 2025 Jan 15;231(4):e650–8. doi: 10.1093/infdis/jiaf033 (PMC11998555; doi:10.1093/infdis/jiaf033)
Supplement: jiaf033_Supplementary_Data [file jiaf033_supplementary_data.zip › Additional-file-2-tableS2.docx]

**Additional file 2:**

Table S2. The number of infected individuals for each of the 14 high-risk HPV types

| **HPV type** |  | **Negative** | **Positive** |
| --- | --- | --- | --- |
|  | **N (total)** | **N (%)** | |
| **HPV type 16** | 4076 | 3906 (95.83%) | 170 (4.17%) |
| **HPV type 18** | 4076 | 3991 (97.91%) | 85 (2.09%) |
| **HPV type 31** | 4076 | 4004 (98.23%) | 72 (1.77%) |
| **HPV type 33** | 4076 | 4047 (99.29%) | 29 (0.71%) |
| **HPV type 35** | 4076 | 3986 (97.79%) | 90 (2.21%) |
| **HPV type 39** | 4076 | 3960 (97.15%) | 116 (2.85%) |
| **HPV type 45** | 4076 | 3991 (97.91%) | 85 (2.09%) |
| **HPV type 51** | 4076 | 3932 (96.47%) | 144 (3.53%) |
| **HPV type 52** | 4076 | 3925 (96.30%) | 151 (3.70%) |
| **HPV type 56** | 4076 | 3994 (97.99%) | 82 (2.01%) |
| **HPV type 58** | 4076 | 3995 (98.01%) | 81 (1.99%) |
| **HPV type 59** | 4076 | 3961 (97.18%) | 115 (2.82%) |
| **HPV type 66** | 4076 | 3959 (97.13%) | 117 (2.87%) |
| **HPV type 68** | 4076 | 4002 (98.18%) | 74 (1.82%) |

Abbreviation: HPV: human papillomavirus.
