# Supplementary material for: Association Between Herpes Simplex Virus Type 2 and High-Risk Human Papillomavirus Infections: A Population Study of the National Health and Nutrition Examination Survey, 2009–2016
Source: J Infect Dis. 2025 Jan 15;231(4):e650–8. doi: 10.1093/infdis/jiaf033 (PMC11998555; doi:10.1093/infdis/jiaf033)
Supplement: jiaf033_Supplementary_Data [file jiaf033_supplementary_data.zip › Additional-file-3-tableS3.docx]

**Additional file 3:**

Table S3. The number of cervical cancer cases by HSV-II infection status.

| **HSV-II** | **Negative** | **Positive** | **Standardize diff.** | **P-value** |
| --- | --- | --- | --- | --- |
| **Cervical Cancer** |  |  | 0.13 (0.06, 0.20) | <0.001 |
| **No** | 2952 (99.09%) | 961 (97.37%) |  |  |
| **Yes** | 27 (0.91%) | 26 (2.63%) |  |  |

Abbreviation: HSV-II: Herpes Simplex Virus II.
